# 2,3-Diphenyl-2,3,5,6-tetra­hydro-4*H*-1,3-thia­zin-4-one

**DOI:** 10.1107/S1600536814000324

**Published:** 2014-01-15

**Authors:** Hemant P. Yennawar, Lee J. Silverberg

**Affiliations:** a Department of Chemistry, Pennsylvania State University, University Park, PA 16802, USA; b Pennsylvania State University, Schuylkill Campus, 200 University Drive, Schuylkill Haven, PA 17972, USA

## Abstract

The six-membered thia­zine ring in the title compound, C_16_H_15_NOS, adopts a half-chair conformation, with the S atom forming the back of the chair. The base of the chair has a slight twist reflected in the r.m.s. deviation (0.0756 Å) of those five atoms from the plane defined by them. The phenyl substituents are almost perpendicular to each other [dihedral angle 87.06 (9)°]. In the crystal, mol­ecules are linked into chains parallel to the *c* axis through C—H⋯O inter­actions.

## Related literature   

For a review of 1,3-thia­zin-4-ones, see: Ryabukhin *et al.* (1996 ▶). For an unsuccessful attempt to make the title compound, see: Surrey *et al.* (1958 ▶). For applications of 2,4,6-tripropyl-1,3,5,2,4,6-trioxatri­phospho­rinane-2,4,6-trioxide (T3P) in the synthesis of amide bonds and heterocycles, see: Dunetz *et al.* (2011 ▶); Unsworth *et al.* (2013 ▶). For the synthesis and structures of related compounds, see: Yennawar *et al.* (2013 ▶); Yennawar & Silverberg (2013 ▶).
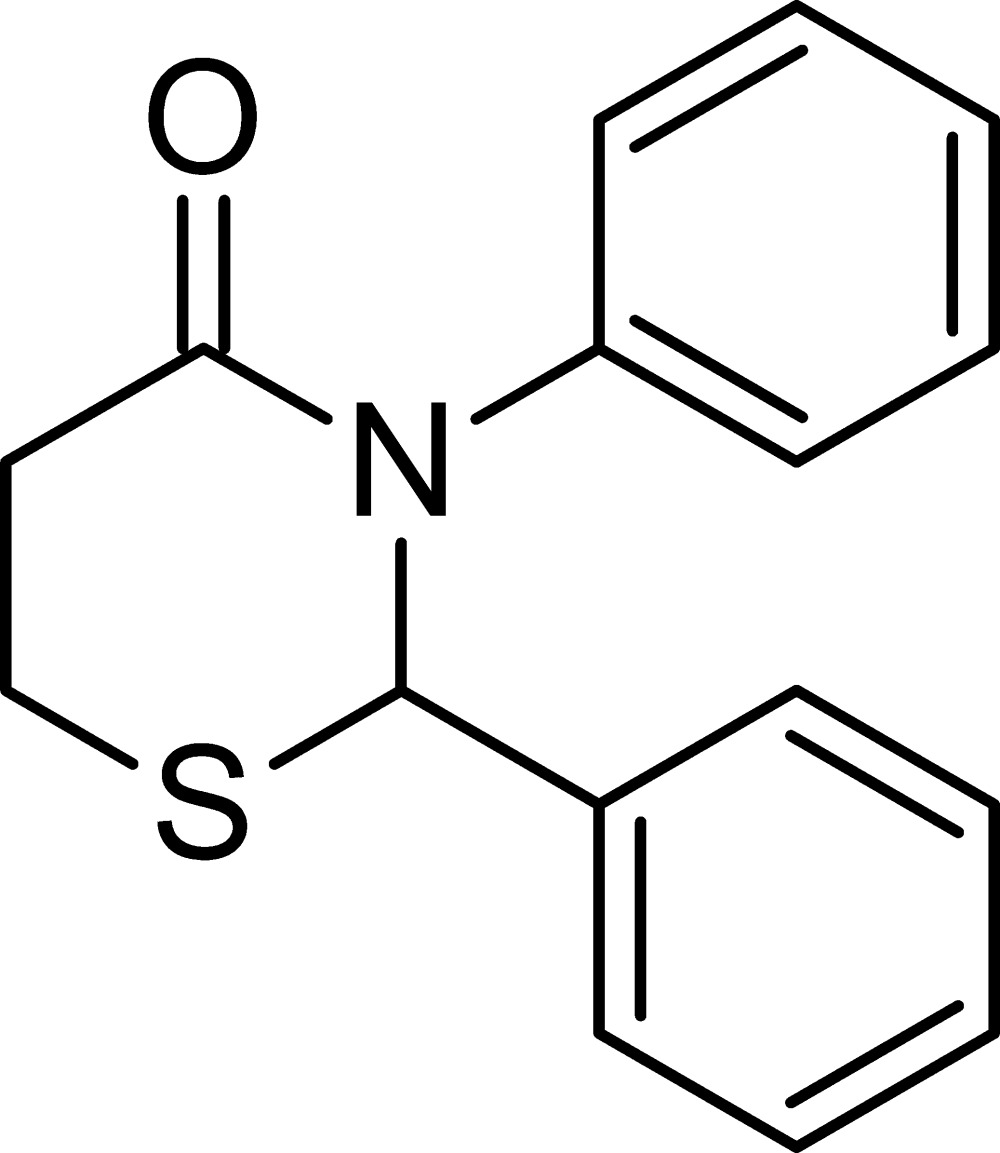


## Experimental   

### 

#### Crystal data  

C_16_H_15_NOS
*M* *_r_* = 269.35
Monoclinic, 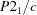

*a* = 13.745 (3) Å
*b* = 8.240 (2) Å
*c* = 12.151 (3) Å
β = 100.079 (6)°
*V* = 1355.0 (6) Å^3^
*Z* = 4
Mo *K*α radiation
μ = 0.23 mm^−1^
*T* = 298 K
0.20 × 0.18 × 0.07 mm

#### Data collection  

Bruker SMART APEX CCD diffractometer
Absorption correction: multi-scan (*SADABS*; Bruker, 2001 ▶) *T*_min_ = 0.956, *T*_max_ = 0.984
12350 measured reflections
3342 independent reflections
2558 reflections with *I* > 2σ(*I*)
*R*_int_ = 0.041

#### Refinement  

*R*[*F* ^2^ > 2σ(*F* ^2^)] = 0.061
*wR*(*F* ^2^) = 0.140
*S* = 1.13
3342 reflections
172 parameters
H-atom parameters not refined
Δρ_max_ = 0.31 e Å^−3^
Δρ_min_ = −0.18 e Å^−3^

### 

Data collection: *SMART* (Bruker, 2001 ▶); cell refinement: *SAINT* (Bruker, 2001 ▶); data reduction: *SAINT* (Bruker, 2001 ▶); program(s) used to solve structure: *SHELXS97* (Sheldrick, 2008 ▶); program(s) used to refine structure: *SHELXL97* (Sheldrick, 2008 ▶); molecular graphics: *SHELXTL* (Sheldrick, 2008 ▶); software used to prepare material for publication: *WinGX* (Farrugia, 2012 ▶).

## Supplementary Material

Crystal structure: contains datablock(s) I. DOI: 10.1107/S1600536814000324/fy2109sup1.cif

Structure factors: contains datablock(s) I. DOI: 10.1107/S1600536814000324/fy2109Isup2.hkl

Supporting information file. DOI: 10.1107/S1600536814000324/fy2109Isup3.mol

Supporting information file. DOI: 10.1107/S1600536814000324/fy2109Isup4.cml

CCDC reference:

Additional supporting information:  crystallographic information; 3D view; checkCIF report

## Figures and Tables

**Table 1:** Hydrogen-bond geometry (Å, °)

*D*—H⋯*A*	*D*—H	H⋯*A*	*D*⋯*A*	*D*—H⋯*A*
C4—H4⋯O1^i^	0.98	2.33	3.265 (3)	159

Symmetry code: (i) 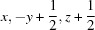.

**Fig. 1.**
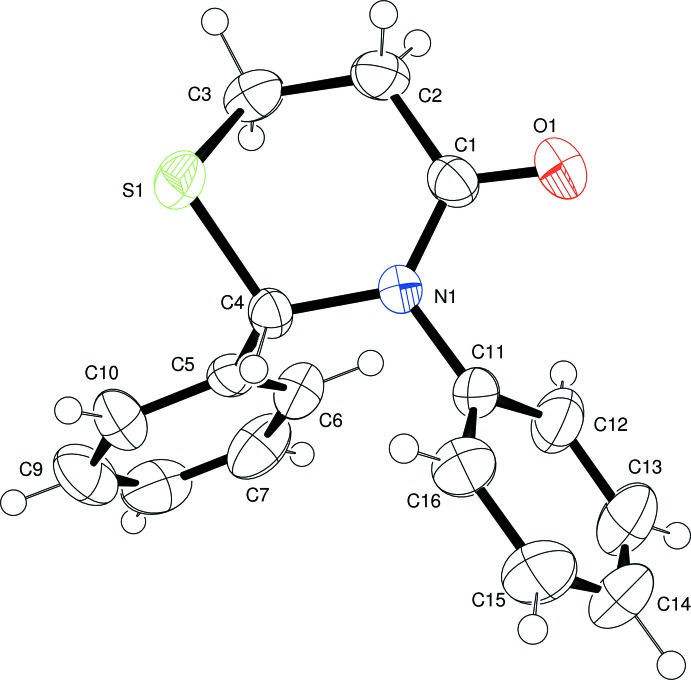
ORTEP view of the title comound. Displacement ellipsoids were drawn at the 50% probability level.

**Fig. 2.**
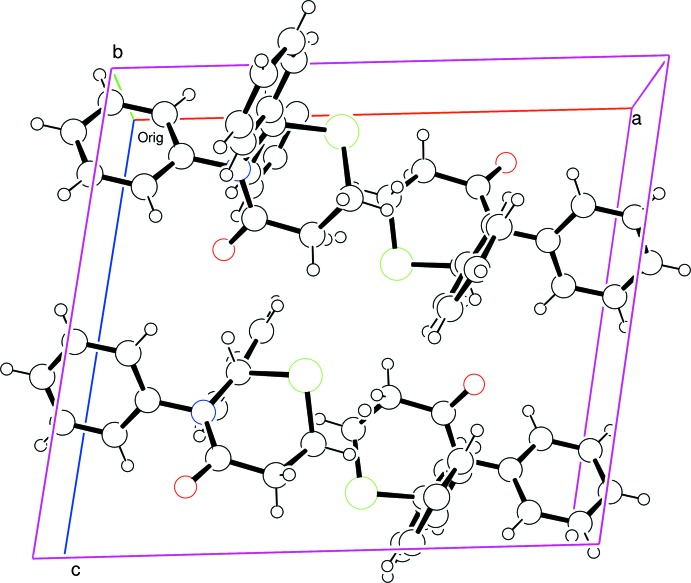
Crystal packing.

## supplementary crystallographic information

### 1. Comment

The 2,3,5,6-tetrahydro-1,3-thiazin-4-ones are an important class of heterocycle with substantial biological activity (Ryabukhin *et al.*, 1996). Surrey has reported that the title compound could not be prepared by condensation of *N*-benzylideneaniline with 3-mercaptopropionic acid in refluxing benzene, unlike when the imine was *N*-alkyl (Surrey *et al.*, 1958). Here we report the synthesis of the novel title molecule by condensation of *N*-benzylideneaniline with 3-mercaptopropionic acid in the presence of 2,4,6-tripropyl-1,3,5,2,4,6-trioxatriphosphorinane-2,4,6-trioxide (T3P) and pyridine (Dunetz *et al.*, 2011). We have recently reported the syntheses of 2-(3-nitrophenyl)-3-phenyl-2,3-dihydro-4*H*-1,3-benzothiazin-4-one (Yennawar *et al.*, 2013) and 6,7-diphenyl-5-thia-7-azaspiro[2.6]nonan-8-one (Yennawar & Silverberg, 2013) by this method. A similar preparation of a 2,3-dialkyl-2,3-dihydro-1,3-benzothiazin-4-one was also recently reported (Unsworth *et al.*, 2013).

### 2. Experimental

A two-necked 25 ml roundbottom flask was oven-dried, cooled under N_2_, and charged with a stir bar and *N*-benzylideneaniline (1.087 g, 6 mmol). Tetrahydrofuran (2.3 ml) was added, the solid dissolved, and the solution was stirred. Pyridine (1.95 ml, 24 mmol) was added and then 3-mercaptopropionic acid (0.523 ml, 6 mmol) was added. Finally, 2,4,6-tripropyl-1,3,5,2,4,6-trioxatriphosphorinane-2,4,6-trioxide in 2-methyltetrahydrofuran (50 weight percent; 7.1 ml, 12 mmol) was added. The reaction was stirred at room temperature for 23 h, then poured into a separatory funnel with dichloromethane (20 ml). The mixture was washed with water (10 ml). The aqueous was then extracted twice with dichloromethane (10 ml each). The organics were combined and washed with saturated sodium bicarbonate (10 ml) and saturated sodium chloride (10 ml). The organic was dried over sodium sulfate, concentrated *in vacuo* and chromatographed on 30 g flash silica gel, eluting with mixtures of ethyl acetate and hexanes (20% to 100% ethyl acetate). The product eluted with 60–100% EtOAc/hexanes and was concentrated *in vacuo* to pale yellow viscous oil (1.3720 g). Recrystallization from ethanol gave white solid (0.7669 g, 47.5%). m.p.: 95–96.5°C. R_f_ = 0.32 (50% EtOAc/hexanes). Crystals for X-ray crystallography were grown by dissolving the solid in ethanol, adding some water, and then allowing slow evaporation to occur. After crystals grew, the supernatant was decanted off, and the crystals were rinsed twice with 95% ethanol.

### 3. Refinement

The C–bound H atoms were geometrically placed with C—H = 0.93–0.97 Å, and refined as riding with *U*_iso_(H) = 1.2U_eq_(C).

### Crystal data

**Table:**

C_16_H_15_NOS	*F*(000) = 568
*M**_r_* = 269.35	*D*_x_ = 1.320 Mg m^−^^3^
Monoclinic, *P*2_1_/*c*	Melting point: 369 K
Hall symbol: -P 2ybc	Mo *K*α radiation, λ = 0.71073 Å
*a* = 13.745 (3) Å	Cell parameters from 2176 reflections
*b* = 8.240 (2) Å	θ = 2.9–24.6°
*c* = 12.151 (3) Å	µ = 0.23 mm^−^^1^
β = 100.079 (6)°	*T* = 298 K
*V* = 1355.0 (6) Å^3^	Block, colorless
*Z* = 4	0.20 × 0.18 × 0.07 mm

### Data collection

**Table:**

Bruker SMART APEX CCD diffractometer	3342 independent reflections
Radiation source: fine-focus sealed tube	2558 reflections with *I* > 2σ(*I*)
Parallel, graphite monochromator	*R*_int_ = 0.041
Detector resolution: 8.34 pixels mm^-1^	θ_max_ = 28.3°, θ_min_ = 2.9°
φ and ω scans	*h* = −18→18
Absorption correction: multi-scan (*SADABS*; Bruker, 2001)	*k* = −10→10
*T*_min_ = 0.956, *T*_max_ = 0.984	*l* = −16→15
12350 measured reflections	

### Refinement

**Table:**

Refinement on *F*^2^	Primary atom site location: structure-invariant direct methods
Least-squares matrix: full	Secondary atom site location: difference Fourier map
*R*[*F*^2^ > 2σ(*F*^2^)] = 0.061	Hydrogen site location: inferred from neighbouring sites
*wR*(*F*^2^) = 0.140	H-atom parameters not refined
*S* = 1.13	*w* = 1/[σ^2^(*F*_o_^2^) + (0.0575*P*)^2^ + 0.2756*P*] where *P* = (*F*_o_^2^ + 2*F*_c_^2^)/3
3342 reflections	(Δ/σ)_max_ = 0.001
172 parameters	Δρ_max_ = 0.31 e Å^−^^3^
0 restraints	Δρ_min_ = −0.18 e Å^−^^3^

### Special details

**Table:**

Geometry. All e.s.d.'s (except the e.s.d. in the dihedral angle between two l.s. planes) are estimated using the full covariance matrix. The cell e.s.d.'s are taken into account individually in the estimation of e.s.d.'s in distances, angles and torsion angles; correlations between e.s.d.'s in cell parameters are only used when they are defined by crystal symmetry. An approximate (isotropic) treatment of cell e.s.d.'s is used for estimating e.s.d.'s involving l.s. planes.
Refinement. Refinement of *F*^2^ against ALL reflections. The weighted *R*-factor *wR* and goodness of fit *S* are based on *F*^2^, conventional *R*-factors *R* are based on *F*, with *F* set to zero for negative *F*^2^. The threshold expression of *F*^2^ > σ(*F*^2^) is used only for calculating *R*-factors(gt) *etc*. and is not relevant to the choice of reflections for refinement. *R*-factors based on *F*^2^ are statistically about twice as large as those based on *F*, and *R*- factors based on ALL data will be even larger.

### Fractional atomic coordinates and isotropic or equivalent isotropic displacement parameters (Å^2^)

**Table:**

	*x*	*y*	*z*	*U*_iso_*/*U*_eq_	
C1	0.70658 (15)	0.2133 (3)	0.70023 (17)	0.0367 (5)	
C2	0.59588 (16)	0.2058 (3)	0.66146 (19)	0.0443 (5)	
H2A	0.5705	0.3158	0.6600	0.053*	
H2B	0.5838	0.1664	0.5851	0.053*	
C3	0.53595 (16)	0.1031 (3)	0.72800 (19)	0.0468 (6)	
H3A	0.5481	−0.0110	0.7162	0.056*	
H3B	0.4661	0.1239	0.7035	0.056*	
C4	0.69656 (14)	0.0797 (2)	0.88595 (16)	0.0306 (4)	
H4	0.7313	0.1214	0.9577	0.037*	
C5	0.70157 (14)	−0.1039 (2)	0.89528 (16)	0.0317 (4)	
C6	0.73750 (16)	−0.2034 (3)	0.8207 (2)	0.0433 (5)	
H6	0.7584	−0.1587	0.7585	0.052*	
C7	0.74288 (18)	−0.3710 (3)	0.8377 (3)	0.0586 (7)	
H7	0.7670	−0.4376	0.7868	0.070*	
C8	0.71254 (19)	−0.4369 (3)	0.9293 (3)	0.0625 (8)	
H8	0.7166	−0.5484	0.9411	0.075*	
C9	0.6762 (2)	−0.3388 (3)	1.0037 (2)	0.0606 (7)	
H9	0.6550	−0.3840	1.0655	0.073*	
C10	0.67093 (18)	−0.1745 (3)	0.98732 (19)	0.0475 (6)	
H10	0.6465	−0.1091	1.0386	0.057*	
C11	0.85580 (14)	0.1465 (3)	0.82879 (18)	0.0367 (5)	
C12	0.91089 (17)	0.0689 (3)	0.7593 (2)	0.0535 (6)	
H12	0.8796	0.0199	0.6936	0.064*	
C13	1.0121 (2)	0.0647 (4)	0.7881 (3)	0.0781 (9)	
H13	1.0492	0.0118	0.7420	0.094*	
C14	1.0589 (2)	0.1379 (5)	0.8843 (3)	0.0823 (11)	
H14	1.1274	0.1356	0.9028	0.099*	
C15	1.0044 (2)	0.2145 (4)	0.9531 (3)	0.0749 (9)	
H15	1.0362	0.2637	1.0185	0.090*	
C16	0.90219 (17)	0.2192 (3)	0.9258 (2)	0.0524 (6)	
H16	0.8653	0.2711	0.9727	0.063*	
N1	0.74904 (11)	0.14718 (19)	0.79997 (13)	0.0316 (4)	
O1	0.75623 (12)	0.2813 (2)	0.64049 (14)	0.0575 (5)	
S1	0.57039 (4)	0.15171 (7)	0.87256 (5)	0.04271 (18)	

### Atomic displacement parameters (Å^2^)

**Table:**

	*U*^11^	*U*^22^	*U*^33^	*U*^12^	*U*^13^	*U*^23^
C1	0.0418 (11)	0.0387 (11)	0.0300 (10)	0.0028 (9)	0.0075 (9)	0.0028 (9)
C2	0.0424 (12)	0.0530 (13)	0.0352 (12)	0.0065 (10)	0.0006 (9)	0.0050 (10)
C3	0.0327 (11)	0.0543 (13)	0.0510 (14)	0.0016 (10)	0.0008 (10)	−0.0004 (11)
C4	0.0321 (10)	0.0345 (10)	0.0260 (9)	−0.0025 (8)	0.0073 (8)	−0.0002 (8)
C5	0.0271 (9)	0.0337 (10)	0.0324 (10)	−0.0023 (7)	−0.0001 (8)	0.0023 (8)
C6	0.0378 (11)	0.0417 (12)	0.0525 (14)	−0.0020 (9)	0.0138 (10)	−0.0028 (10)
C7	0.0433 (13)	0.0410 (13)	0.094 (2)	0.0012 (10)	0.0176 (13)	−0.0159 (14)
C8	0.0509 (14)	0.0345 (12)	0.096 (2)	−0.0045 (11)	−0.0033 (14)	0.0155 (14)
C9	0.0751 (19)	0.0483 (15)	0.0552 (16)	−0.0134 (13)	0.0024 (14)	0.0187 (13)
C10	0.0596 (15)	0.0456 (13)	0.0380 (12)	−0.0084 (11)	0.0105 (11)	0.0057 (10)
C11	0.0304 (10)	0.0388 (11)	0.0416 (12)	−0.0023 (8)	0.0079 (8)	0.0100 (9)
C12	0.0406 (13)	0.0620 (16)	0.0622 (16)	−0.0002 (11)	0.0210 (12)	0.0015 (13)
C13	0.0433 (15)	0.101 (2)	0.096 (2)	0.0096 (15)	0.0308 (16)	0.016 (2)
C14	0.0302 (13)	0.116 (3)	0.100 (3)	−0.0001 (15)	0.0103 (16)	0.032 (2)
C15	0.0471 (16)	0.099 (2)	0.071 (2)	−0.0182 (16)	−0.0107 (14)	0.0139 (17)
C16	0.0413 (13)	0.0632 (16)	0.0502 (14)	−0.0058 (11)	0.0010 (11)	0.0036 (12)
N1	0.0297 (8)	0.0362 (9)	0.0296 (8)	−0.0012 (7)	0.0073 (6)	0.0046 (7)
O1	0.0549 (10)	0.0746 (12)	0.0447 (10)	−0.0011 (9)	0.0138 (8)	0.0237 (9)
S1	0.0376 (3)	0.0476 (3)	0.0466 (3)	0.0076 (2)	0.0176 (2)	0.0004 (3)

### Geometric parameters (Å, º)

**Table:**

C1—O1	1.217 (2)	C7—H7	0.9300
C1—N1	1.363 (3)	C8—C9	1.370 (4)
C1—C2	1.513 (3)	C8—H8	0.9300
C2—C3	1.511 (3)	C9—C10	1.368 (3)
C2—H2A	0.9700	C9—H9	0.9300
C2—H2B	0.9700	C10—H10	0.9300
C3—S1	1.783 (2)	C11—C16	1.376 (3)
C3—H3A	0.9700	C11—C12	1.385 (3)
C3—H3B	0.9700	C11—N1	1.448 (2)
C4—N1	1.478 (2)	C12—C13	1.374 (4)
C4—C5	1.518 (3)	C12—H12	0.9300
C4—S1	1.813 (2)	C13—C14	1.371 (5)
C4—H4	0.9800	C13—H13	0.9300
C5—C6	1.377 (3)	C14—C15	1.370 (5)
C5—C10	1.390 (3)	C14—H14	0.9300
C6—C7	1.396 (3)	C15—C16	1.387 (4)
C6—H6	0.9300	C15—H15	0.9300
C7—C8	1.368 (4)	C16—H16	0.9300
			
O1—C1—N1	121.12 (19)	C7—C8—H8	120.0
O1—C1—C2	118.13 (19)	C9—C8—H8	120.0
N1—C1—C2	120.75 (18)	C10—C9—C8	120.3 (2)
C3—C2—C1	117.96 (18)	C10—C9—H9	119.9
C3—C2—H2A	107.8	C8—C9—H9	119.9
C1—C2—H2A	107.8	C9—C10—C5	121.0 (2)
C3—C2—H2B	107.8	C9—C10—H10	119.5
C1—C2—H2B	107.8	C5—C10—H10	119.5
H2A—C2—H2B	107.2	C16—C11—C12	120.2 (2)
C2—C3—S1	109.00 (16)	C16—C11—N1	120.3 (2)
C2—C3—H3A	109.9	C12—C11—N1	119.6 (2)
S1—C3—H3A	109.9	C13—C12—C11	119.6 (3)
C2—C3—H3B	109.9	C13—C12—H12	120.2
S1—C3—H3B	109.9	C11—C12—H12	120.2
H3A—C3—H3B	108.3	C14—C13—C12	120.6 (3)
N1—C4—C5	113.97 (16)	C14—C13—H13	119.7
N1—C4—S1	113.02 (13)	C12—C13—H13	119.7
C5—C4—S1	111.29 (13)	C15—C14—C13	119.9 (3)
N1—C4—H4	105.9	C15—C14—H14	120.1
C5—C4—H4	105.9	C13—C14—H14	120.1
S1—C4—H4	105.9	C14—C15—C16	120.4 (3)
C6—C5—C10	118.4 (2)	C14—C15—H15	119.8
C6—C5—C4	124.14 (19)	C16—C15—H15	119.8
C10—C5—C4	117.46 (18)	C11—C16—C15	119.4 (3)
C5—C6—C7	120.5 (2)	C11—C16—H16	120.3
C5—C6—H6	119.8	C15—C16—H16	120.3
C7—C6—H6	119.8	C1—N1—C11	118.34 (16)
C8—C7—C6	119.8 (2)	C1—N1—C4	126.35 (16)
C8—C7—H7	120.1	C11—N1—C4	115.27 (15)
C6—C7—H7	120.1	C3—S1—C4	95.72 (10)
C7—C8—C9	120.0 (2)		
			
O1—C1—C2—C3	172.2 (2)	C13—C14—C15—C16	−0.3 (5)
N1—C1—C2—C3	−8.5 (3)	C12—C11—C16—C15	0.2 (4)
C1—C2—C3—S1	49.0 (2)	N1—C11—C16—C15	178.7 (2)
N1—C4—C5—C6	−9.7 (3)	C14—C15—C16—C11	−0.1 (4)
S1—C4—C5—C6	119.51 (19)	O1—C1—N1—C11	−5.6 (3)
N1—C4—C5—C10	167.98 (17)	C2—C1—N1—C11	175.19 (19)
S1—C4—C5—C10	−62.8 (2)	O1—C1—N1—C4	172.0 (2)
C10—C5—C6—C7	−0.1 (3)	C2—C1—N1—C4	−7.2 (3)
C4—C5—C6—C7	177.6 (2)	C16—C11—N1—C1	122.7 (2)
C5—C6—C7—C8	−0.2 (4)	C12—C11—N1—C1	−58.8 (3)
C6—C7—C8—C9	0.5 (4)	C16—C11—N1—C4	−55.1 (3)
C7—C8—C9—C10	−0.6 (4)	C12—C11—N1—C4	123.4 (2)
C8—C9—C10—C5	0.3 (4)	C5—C4—N1—C1	108.3 (2)
C6—C5—C10—C9	0.0 (3)	S1—C4—N1—C1	−20.1 (2)
C4—C5—C10—C9	−177.9 (2)	C5—C4—N1—C11	−74.1 (2)
C16—C11—C12—C13	0.2 (4)	S1—C4—N1—C11	157.54 (14)
N1—C11—C12—C13	−178.4 (2)	C2—C3—S1—C4	−64.60 (17)
C11—C12—C13—C14	−0.6 (4)	N1—C4—S1—C3	51.31 (16)
C12—C13—C14—C15	0.7 (5)	C5—C4—S1—C3	−78.43 (16)

### Hydrogen-bond geometry (Å, º)

**Table:**

*D*—H···*A*	*D*—H	H···*A*	*D*···*A*	*D*—H···*A*
C4—H4···O1^i^	0.98	2.33	3.265 (3)	159

Symmetry code: (i) *x*, −*y*+1/2, *z*+1/2.
