# 2,3-Diphenyl-2,3,5,6-tetra­hydro-4*H*-1,3-thia­zin-4-one. Corrigendum

**DOI:** 10.1107/S2056989015022963

**Published:** 2015-12-06

**Authors:** Hemant P. Yennawar, Lee J. Silverberg

**Affiliations:** a Department of Chemistry, Pennsylvania State University, University Park, PA 16802, USA; b Pennsylvania State University, Schuylkill Campus, 200 University Drive, Schuylkill Haven, PA 17972, USA

## Abstract

In the paper by Yennawar & Silverberg [*Acta Cryst.* (2014), E**70**, o133], the conformation of the thiazine ring was reported incorrectly.

## Abstract 2

Erratum to *Acta Cryst.* (2014), E**70**, o133.

The authors of Yennawar & Silverberg (2014) ▸ have indicated that the conformation of the thiazine ring in the title compound should be an envelope, with the S atom forming the flap, and not half-chair as originally given.
